# Intravenous Topiramate: Pharmacokinetics in Dogs with Naturally Occurring Epilepsy

**DOI:** 10.3389/fvets.2016.00107

**Published:** 2016-12-05

**Authors:** Irene Vuu, Lisa D. Coles, Patricia Maglalang, Ilo E. Leppik, Greg Worrell, Daniel Crepeau, Usha Mishra, James C. Cloyd, Edward E. Patterson

**Affiliations:** ^1^Center for Orphan Drug Research, University of Minnesota, Minneapolis, MN, USA; ^2^Department of Experimental and Clinical Pharmacology, College of Pharmacy, University of Minnesota, Minneapolis, MN, USA; ^3^College of Science and Engineering, University of Minnesota, Minneapolis, MN, USA; ^4^UMP MINCEP Epilepsy Care, Minneapolis, MN, USA; ^5^Mayo Clinic, Rochester, MN, USA; ^6^College of Veterinary Medicine, University of Minnesota, Saint Paul, MN, USA

**Keywords:** translational, dog, epilepsy, seizures, topiramate, ASD

## Abstract

**Rationale:**

Barriers to developing treatments for human status epilepticus include the inadequacy of experimental animal models. In contrast, naturally occurring canine epilepsy is similar to the human condition and can serve as a platform to translate research from rodents to humans. The objectives of this study were to characterize the pharmacokinetics of an intravenous (IV) dose of topiramate (TPM) in dogs with epilepsy and evaluate its effect on intracranial electroencephalographic (iEEG) features.

**Methods:**

Five dogs with naturally occurring epilepsy were used for this study. Three were getting at least one antiseizure drug as maintenance therapy including phenobarbital (PB). Four (ID 1–4) were used for the 10 mg/kg IV TPM + PO TPM study, and three (ID 3–5) were used for the 20 mg/kg IV TPM study. IV TPM was infused over 5 min at both doses. The animals were observed for vomiting, diarrhea, ataxia, and lethargy. Blood samples were collected at scheduled pre- and post-dose times. Plasma concentrations were measured using a validated high-performance liquid chromatography-mass spectrometry method. Non-compartmental and population compartmental modeling were performed (Phoenix WinNonLin and NLME) using plasma concentrations from all dogs in the study. iEEG was acquired in one dog. The difference between averaged iEEG energy levels at 15 min pre- and post-dose was assessed using a Kruskal–Wallis test.

**Results:**

No adverse events were noted. TPM concentration–time profiles were best fit by a two compartment model. PB co-administration was associated with a 5.6-fold greater clearance and a ~4-fold shorter elimination half-life. iEEG data showed that TPM produced a significant energy increase at frequencies >4 Hz across all 16 electrodes within 15 min of dosing. Simulations suggested that dogs on an enzyme inducer would require 25 mg/kg, while dogs on non-inducing drugs would need 20 mg/kg to attain the target concentration (20–30 μg/mL) at 30 min post-dose.

**Conclusion:**

This study shows that IV TPM has a relatively rapid onset of action, loading doses appear safe, and the presence of PB necessitates a higher dose to attain targeted concentrations. Consequently, it is a good candidate for further evaluation for treatment of seizure emergencies in dogs and people.

## Introduction

Status epilepticus (SE) is defined as a condition characterized by abnormally prolonged seizures that can lead to long-term consequences, including permanent neuronal injury (1). SE has been reported to have an incidence between 2.5 and 59% in dogs with idiopathic epilepsy (2–4), and 32% in dogs with secondary epilepsy (3). In dogs that have had at least one episode of SE, overall mortality rates (primarily from euthanasia) were 32–38% (2, 5). In humans, SE occurs with an incidence between 0.04 and 0.06% in the United States, resulting in an overall mortality rate of 22% (6). While benzodiazepines are the standard first line of care for SE in both dogs and humans (7, 8), approximately one-third of humans fail to respond to first-line therapy (9). There remains a need for safe alternatives for early and rapid first- and/or second-line therapy of SE to reduce the probability of recurring seizures, minimize associated complications, and improve patient outcomes.

One of the barriers to developing new treatments for SE is the experimental model used to find and evaluate investigational therapies. Oftentimes in rodent models, epilepsy is induced by chemical or electrical insult and may not be truly representative of epilepsy pathophysiology (10). Dogs with naturally occurring epilepsy have been proposed as appropriate models to examine new antiepileptic therapies prior to human trials (11). Canine epilepsy is strikingly similar to the human condition in both disease presentation and response to treatment. Holliday et al. demonstrated that intracranial electroencephalograms (EEGs) of dogs and humans during focal onset seizure are indistinguishable (12). Moreover, studies of antiseizure drugs (ASDs), such as fosphenytoin and levetiracetam, have shown comparable efficacy in both dogs and humans for SE (11, 13). Given these similarities, assessing new therapies for SE in dogs will facilitate drug development and increase the chance of successful translation for both canine and human SE.

Among the newer ASDs with injectable formulations, topiramate (TPM) is an attractive candidate for evaluation in the treatment of SE. TPM is the second-generation, broad-spectrum ASD that inhibits of voltage-gated sodium channels and enhances gamma-aminobutyrate (GABA) activity at specific GABA_A_ receptor subtypes (14). TPM also has mechanisms of action that differ from those exhibited by current therapies, including antagonizing AMPA/kainate glutamate receptors, and inhibiting specific carbonic anhydrase isozymes. Specifically in rodent studies of SE and ischemia, TPM has exhibited neuroprotection (15, 16). There are also several clinical reports in which TPM suspensions administered in humans *via* nasogastric tube was associated with seizure cessation in refractory SE. In both adults and children as young as 4.5 months, plasma concentrations of 2–40 μg/mL were associated with resolution of refractory SE (17–23).

Our group has studied the pharmacokinetics (PK) of a novel intravenous (IV) TPM formulation in humans. However, the PK of IV TPM has not been characterized in dogs. Furthermore, while oral TPM might be useful in dogs, there is limited information on oral PK and no information in dogs with naturally occurring epilepsy on antiseizure medications (24). The aims of this study were to (1) characterize TPM PK following an IV and oral dose and (2) simulate doses to attain target concentrations of 20–30 μg/mL, upper range of concentrations that have been associated with efficacy in humans. As an exploratory analysis, we also report the effect of IV TPM on intracranial electroencephalographic (iEEG) features in one dog.

## Materials and Methods

### Study Animals and Safety Monitoring

Five dogs with naturally occurring epilepsy were used in this study. Three of the dogs have uncontrolled seizures despite being on antiseizure maintenance regimens. Approval was obtained through the Institutional Animal Care and Use Committee of the University of Minnesota prior to the initiation of the study. The dogs were housed at the University of Minnesota’s Veterinary College. Each dog was previously implanted with a device which wirelessly transmits iEEG recordings (25, 26). Dogs were monitored throughout the study for vomiting, diarrhea, and lethargy prior to and for 90 min after drug administration, and at each blood sampling time. In the event of a seizure emergency (seizure lasting >5 min) or repetitive seizures (2+ seizures within 1 h, or 3+ seizures within 4 h), the on call veterinarian received an automated text message and confirmed the seizure activity using remote video monitoring. The rescue therapy protocol consisted of midazolam 12 mg administered as a single intramuscular dose.

### Study Drug

For this study, a stable isotope-labeled TPM compound containing six ^13^C, resulting in a mass 6 U greater than the unlabeled molecule was used for the IV formulation (10 mg/mL in 10% Captisol^®^). This formulation was manufactured by the University of Iowa under Good Manufacturing Practices and has been licensed to Ligand/CuRx Pharmaceuticals. Unlabeled TPM tablets (25 mg) purchased from the University of Minnesota Veterinary Pharmacy (Cipla USA, Inc.) were used for the oral treatment arm. Using a labeled IV formulation and non-labeled oral tablets allowed us to simultaneously administer both formulations and characterize TPM PK by each route. This approach also reduces interoccasion variability caused by dosing on different days and/or times (27).

### Dose Rationale

Based on reports of doses associated with efficacy in human SE (2–40 μg/mL), we aimed for a plasma TPM concentration on the higher end of the range (20–30 μg/mL) for a higher likelihood of efficacy without risking safety (17–23). A previous single IV dose study in one dog reported TPM concentrations from which we calculated an apparent volume of distribution (Vd) of 0.6 L/kg (24). Using this Vd, we estimated that IV doses of 10 and 20 mg/kg would produce initial concentrations (C_0_) of ~16 and 32 μg/mL, respectively.

### Study Design

For low dose IV/oral TPM study, four dogs were used in this study (ID 1–4; Table 1). Two of the four dogs were on ASD maintenance regimen including phenobarbital (PB). Each dog was fasted overnight prior to receiving a 10 mg/kg dose of stable-labeled IV TPM infused over 5 min. One hour following the IV bolus, each dog also received a 5 mg/kg dose of unlabeled oral TPM. This delay in oral administration was by design to allow evaluation of the IV dose on iEEG for 1 h after dosing. Each dog was fed no sooner than 2 h after the oral dose. Blood samples (~5 mL) were collected from an indwelling catheter prior to dosing and at 0.083, 0.25, 0.5, 0.75, 1, 1.5, 2, 2.5, 3, 4, 6, 8, and 9 h following the IV bolus.

**Table 1:** **Animal demographics at time of study**.

Subject	Age (years)	Gender	Weight (kg)	Breed	Seizure type	Seizure frequency	Co-medications
1	5	Male, intact	33	Coonhound mix	Focal, with generalized seizures	Generalized cluster seizures once every 3 weeks	Levetiracetam, zonisamide, phenobarbital
2	9	Male, neutered	29	Labrador retriever mix	Focal, with generalized seizures	Focal cluster seizures every 2–4 days. With secondarily generalized cluster seizures every 1–2 weeks	Levetiracetam, zonisamide, phenobarbital, potassium bromide
3	3	Male, intact	15	Beagle	N/A	Seizure-free and in remission for 2 years (had one witnessed generalized seizure)	N/A
4	5	Female, spayed	29	Coonhound mix	N/A	Seizure-free for and in remission 2.5 years (had one witnessed generalized seizure)	N/A
5	5	Male, intact	35	Coonhound mix	Focal, with secondary generalized seizures	Single generalized seizures once every 2–3 months	Phenobarbital

#### High-Dose IV TPM Study

Three dogs were used in this study (ID 3–5; Table 1). One dog was on PB maintenance therapy. Each dog was fasted overnight prior to receiving a 20 mg/kg dose of stable-labeled IV TPM infused over 5 min. Blood samples (~5 mL) were collected from an indwelling catheter prior to dosing and at 0.083, 0.25, 0.5, 0.75, 1, 1.5, 2, 2.5, 3, 4, 6, 8, and 9 h following the IV bolus.

#### Diazepam Positive Control

Intravenous diazepam (DZP) (0.5 mg/kg) was administered to two dogs that were having uncontrolled seizures (ID 1 and 2) during an interictal period as a positive control as it has been shown to elicit iEEG change.

### TPM Plasma Measurements

Upon sample collection, blood was placed on ice, and plasma was separated. All samples were immediately frozen (−20°C) until analysis. A high-performance liquid chromatography-mass spectrometry (HPLC-MS) method developed and validated at the Center for Orphan Drug Research was used to measure TPM concentrations in dog plasma. Seven calibration standards (run in triplicate) and nine quality control standards (low, medium, and high run in triplicates) were prepared in plasma. Study, calibration, and quality control samples (250 μL) were extracted using methyl tert-butyl ether. TPM and stable-labeled TPM were analyzed using the Hewlett Packard Agilent 1100 Model G1946 liquid chromatography mass spectrometry detection system and Agilent ChemStation software. The analytes were separated using a Zorbax C18 column (150 mm × 3.0 mm, 3 μm), and the mobile phase consisted of an ammonium acetate buffer and methanol. The quantization was performed using the selective ion monitoring in the negative mode, with deuterated TPM (d10) as the internal standard. The mass-to-charge ratios were 338 and 244 *m*/*z* for TPM and stable-labeled TPM, respectively. The calibration curves were linear (*r*^2^ = 0.998) in the concentration range of 0.05–50 μg/mL for stable-labeled TPM and 0.05–10 μg/mL for TPM in plasma. The limit of detection and quantitation was 0.05 ng/mL and 0.05 μg/mL, respectively. The precision for both TPM and stable-labeled TPM ranged from 3 to 6%, and accuracy values were between 95 and 114% and 86 and 105%, respectively.

### Pharmacokinetic Analysis

Topiramate concentration–time data were analyzed using non-compartmental analysis (Phoenix WinNonLin, version 6.4, Pharsight Corporation, Mountain View, CA, USA). Pharmacokinetic parameter values included maximum concentration (*C*_max_), time at which maximum concentration is achieved (*t*_max_), elimination rate half-life (*t*_1/2_), and the area under the time–concentration curve (AUC_INF_) calculated using the equation $\text{AUC}=\overset{t=\infty }{\underset{t=0}{\int }}\text{Cp}\times dt$ (where Cp is the plasma TPM concentration) and a linear-log trapezoidal method. Oral bioavailability (F%) was calculated using the equation $\text{F}\left(\%\right)=\frac{\text{AUC}\left(\text{oral}\right)\times \text{Dose}\left(\text{IV}\right)}{\text{AUC}\left(\text{IV}\right)\times \text{Dose}\left(\text{oral}\right)}\times 100$. Clearance (CL) and Vd were calculated using the equations $\text{CL}=\frac{\text{Dose}\times \text{F}}{\text{AUC}}$ and CL = *k_e_* × Vd, respectively, where *k_e_* is the elimination rate constant. Concentration–time profiles were created using the GraphPad Prism 7 (Version 7.0a, GraphPad Software, Inc., La Jolla, CA, USA).

Pharmacokinetics parameters were also determined using population compartmental modeling (Phoenix Non-Linear Mixed Effects software, version 1.3, Pharsight Corporation, Mountain View, CA, USA). First-order conditional estimation extended least squares method was used throughout the model building process. One and two compartment models were evaluated. A proportional error model for between subject variability was used. Both additive and multiplicative error models for residual variability were evaluated. The best fit model was determined using visual inspection, goodness of fit plots, weighted residual plots, weighted sum of squared residuals, Akaike’s Information Criterion, and precision of model parameters.

The presence of a CYP3A4-inducing co-medication (such as PB) was evaluated as a covariate for its influence on TPM clearance. The relationship of the covariate and TPM clearance was modeled by the equation Cl = tvCl × *e*^dCl^ × *e*^ηCl^, where Cl is the clearance from the central compartment, tvCl is the typical value of the clearance from the population, dCl is the estimated value of the inducer effect, and ηCl is the between-subject variability (BSV) of clearance. A covariate was considered statistically significant if inclusion of the covariate resulted in a decrease in the objective function value (OFV) of at least 6.64 (*p* < 0.01, *x*^2^, degree of freedom = 1). The final model was used to simulate of 5-, 10-, and 15-min infusions IV TPM at doses ranging from 10 to 30 mg/kg.

### Electroencephalographic Analysis

Intracranial electroencephalographic analysis was performed in one dog (ID 3) that was not having uncontrolled seizures (not on co-medications). Sixteen electrode channels were continuously sampled at 399.6 Hz. A bandpass filter was applied to create six frequency bands: delta (1–4 Hz), theta (4–8 Hz), alpha (8–12 Hz), beta (12–25 Hz), low gamma (25–40 Hz), and high gamma (40–120 Hz). In order to evaluate differences in EEG features, energy of each electrode within each frequency band was calculated in 1-s intervals by summing the square of the EEG signal amplitude within the 1-s window. The average energy level was calculated for three 15-min ranges: starting from 15-min pre-dose to dosing, from dosing to 15-min post-dose, and from 15-min post-dose to 30-min post-dose. The difference between averaged energy levels at pre-dose and each post-dose interval were calculated. *p* Values were generated by the Kruskal–Wallis test comparing the averaged energy level from pre-dose to the two averaged energy levels post-dose. While EEG systems were implanted in all dogs, EEG data were not attained for all animals due to electrical malfunctioning of the electrodes and/or data cards as some of the devices had been implanted for up to 5 years.

## Results

### Demographics and Adverse Events

Demographics of the dogs are represented in Table 1. No adverse events were observed for either dose group throughout the course of the study.

### Non-Compartmental PK Analysis

**Figure 1:**
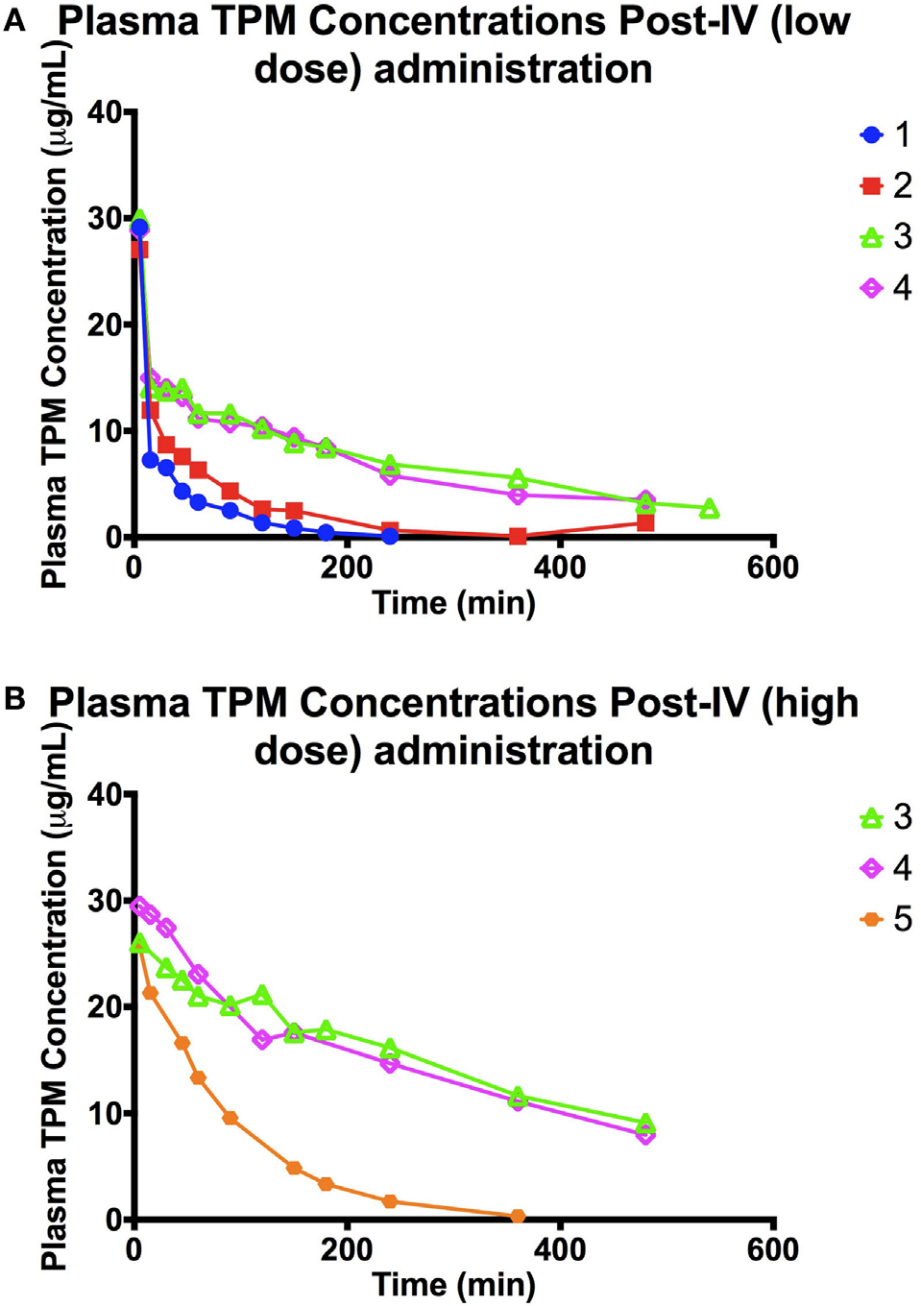
**(A)** Plasma TPM concentration time profile following an intravenous bolus (low dose: 10 mg/kg) of stable-labeled TPM. **(B)** Plasma TPM concentration time profile following an intravenous bolus (high dose: 20 mg/kg) of stable-labeled TPM.

The concentration–time profiles of plasma ^13^C-TPM following the low- and high-dose IV infusions are shown in Figure 1. Pharmacokinetic parameter estimates using non-compartmental analysis are summarized in Table 2. TPM clearance was greater and elimination half-life shorter in dogs receiving chronic PB. The clearance was 0.5–0.7 versus 0.1 L/h/kg and elimination half-life 0.5–1 versus 3.7–5 h in dogs with and without PB, respectively, suggesting hepatic enzyme induction by PB. Clearance, volume of distribution, and elimination half-life were similar for both dose groups studied. AUC_INF_ approximately doubled as dose doubled suggesting dose-proportional PK.

**Table 2:** **Pharmacokinetic parameter values estimated from non-compartmental analysis after an intravenous bolus of stable-labeled TPM**.

ID	Group	*t*_1/2_ (h)	C_1_ (μg/mL)	AUC_INF__obs (μg × h/mL)	V_obs (L/kg)	Cl_obs (L/h/kg)
1	LOW	0.47	29.2	13.7	0.50	0.73
2	LOW	0.75	27.1	20.6	0.53	0.49
3	LOW	3.71	30	83.5	0.64	0.12
4	LOW	4.05	28.9	101	0.58	0.1
3	HIGH	4.99	26.1	194	0.74	0.1
4	HIGH	4.52	29.5	176	0.74	0.11
5	HIGH	0.95	25.7	38.4	0.71	0.52

*LOW = 10 mg/kg dose; HIGH = 20 mg/kg dose; t_1/2_, elimination half-life; C_1_, first measured concentration; AUC_INF__obs, observed area under the curve from time 0 to infinity; V_obs, observed volume of distribution; Cl_obs, observed clearance*.

**Figure 2:**
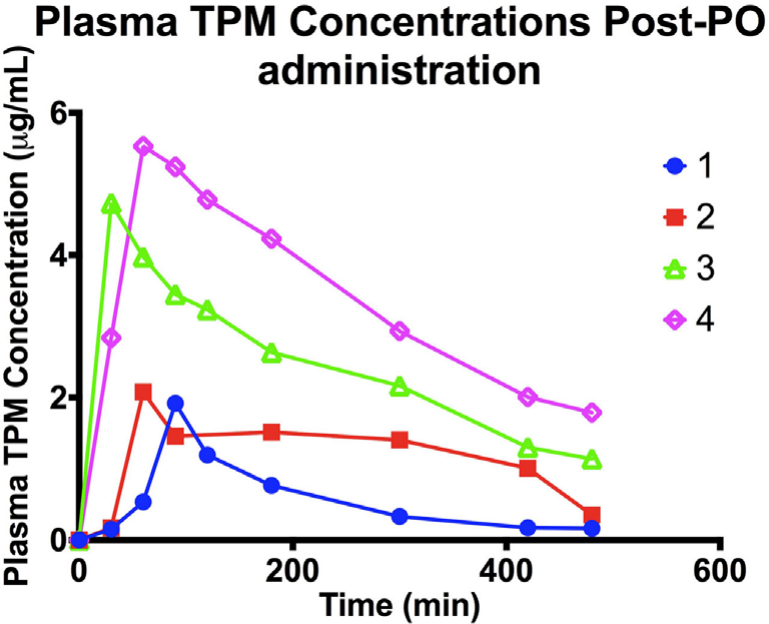
**Plasma TPM concentration time profile following an oral dose (5 mg/kg) of TPM**.

Plasma TPM concentration–time profiles following oral administration are depicted in Figure 2. *C*_max_ following oral administration ranged between 1.9 and 2 μg/mL at 1–1.5 h (*T*_max_), with a *t*_1/2_ between 1.7 and 2 h in the two dogs on PHB. In the two dogs not on PB, a *C*_max_ of 4.7–5.5 μg/mL at 0.5–1 h was observed, with a t_1/2_ of 4 h. Individual oral bioavailability ranged between 61 and 102%. These results are summarized in Table 3. Similar to the IV administration, the two dogs on PHB exhibited higher clearance rates, and consequently, shorter half-lives compared to the two dogs not on PB.

**Table 3:** **Pharmacokinetic parameter values estimated from non-compartmental analysis after an oral dose (5 mg/kg) of unlabeled TPM**.

ID	*t*_1/2_ (h)	*T*_max_ (min)	*C*_max_ (μg/mL)	AUC_INF__obs (μg × h/mL)	V_obs (L/kg)	Cl_obs (L/h/kg)	F (%)
1	2.02	90	1.92	4.73	2.13	0.73	69.2
2	1.66	60	2.08	10.5	1.16	0.49	102
3	3.98	30	4.73	25.7	0.69	0.12	61.7
4	4.08	60	5.53	36.9	0.58	0.1	73.0

*Volume and clearance were adjusted for bioavailability*.

*t_1/2_, elimination half-life; T_max_, time at peak concentration; C_max_, peak concentration, AUC_INF__obs, observed area under the curve from time 0 to infinity; V_obs, observed volume of distribution; Cl_obs, observed clearance; F, bioavailability*.

### Compartmental PK Analysis

A two compartment model with first-order elimination best fit the TPM concentration data following IV administration (Figure S1 in Supplementary Material). Parameter estimates are provided in Table S1 in Supplementary Material. A systematic bias in clearance based on dose was observed. The inclusion of whether the dog was on an enzyme-inducing co-medication as a covariate resulted in a decrease in the OFV from the base model (difference in OFV = 25) and an improvement in the goodness of fit plots and precision of parameter estimates. Therefore, the effect of an enzyme inducer on clearance was included in the final model. The presence of PB is estimated to affect TPM clearance by a factor of 5.64. Except for peripheral compartment clearance, all model-fitted parameters were estimated with good precision with all coefficients of variation below 25%. A multiplicative error model best described the residual error with an estimate of 15%, which is consistent with analytical error. Visual predictive check plots (Figure S2 in Supplementary Material) illustrated the observed data percentiles fall within the 90% (5–95%) model-predicted intervals.

### Simulation Analysis

**Figure 3:**
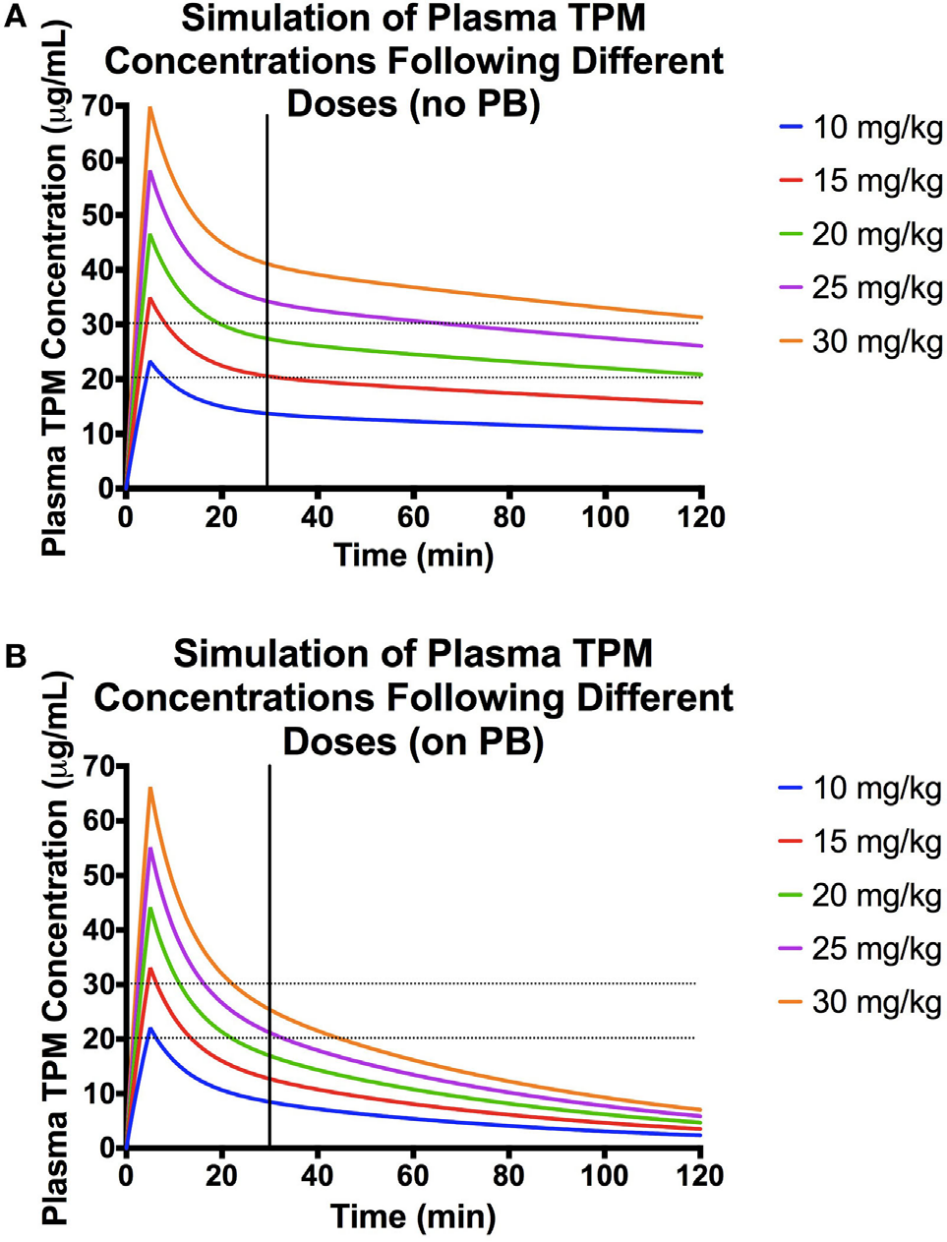
**Population pharmacokinetic parameters used for the simulations: volume of distribution (Vd) of central compartment = 376 mL/kg, Vd of peripheral compartment = 298 mL/kg, clearance (CL) from central compartment = 1.84 mg/(kg × min), CL from peripheral compartment = 21 mL/(kg × min), and effect of PB presence on CL = 1.73**. **(A)** Simulations of plasma concentration–time profiles following 5-min infusions of different doses in a dog not on enzyme-inducing co-medications. Here, the desired range is represented by the black dashed lines, and the black vertical line denotes 30 min post-dose. **(B)** Simulations of plasma concentration–time profiles following 5-min infusions of different doses in a dog on enzyme-inducing co-medications. Here, the desired range is represented by the black dashed lines, and the black vertical line denotes 30 min post-dose.

Using the final model above, various infusion rates and doses were simulated (Figure 3). For dogs not on enzyme-inducing co-medications, simulated time-concentration profiles suggest that a 5-min infusion of 20 mg/kg would achieve target concentration range of 20–30 μg/mL at 30 min post-dose. However, in dogs on enzyme-inducing co-medications, a dose between 25 and 30 mg/kg infused over 5 min would be required to attain the same target range.

### EEG Analysis

In one dog (ID 3), IV TPM produced EEG changes shortly after the infusion, which continued in the subsequent 40–60 min (Figure S3 in Supplementary Material). Statistically significant positive energy differences in all 6 frequency bands across all 16 channels were seen comparing the pre-dose time (−15–0 min) to both post-dose times (0–15 min and 15–30 min) (Table S2 in Supplementary Material). Detailed energy differences across the 6 frequency bands in each of the 16 channels comparing the pre-dose time to 0–15 min post-dose are shown in Table S3 in Supplementary Material.

## Discussion

This study is unique in that it evaluated the PK of IV and oral TPM in dogs with naturally occurring epilepsy. This is also the first study that reports the absolute bioavailability of oral TPM and the effect of PB on TPM PK in dogs. The oral bioavailability was 62–102% that is similar to what has been reported in humans (28). Furthermore, enzyme induction increases TPM clearance in both humans and dogs. In humans, enzyme-inducing co-medications such as carbamazepine and phenytoin showed a 1.5-fold increase in TPM clearance. In our study, PB increased TPM clearance by 5.6-fold. To explain this discrepancy, Caldwell et al. found that while 82% of TPM is excreted unchanged in the urine in humans, only 28% is excreted unchanged in dogs (29). PB is a known inducer of CYP 3A4, the major enzyme responsible for TPM metabolism. A recent study evaluating the effects of chronic administration of PB on the PK of levetiracetam in dogs with epilepsy found similar results (30). Potential drug–drug interactions should be taken into consideration when dosing TPM in both dogs and humans. Dose adjustments are likely needed when TPM is used in conjunction with chronic enzyme-inducing or enzyme-inhibiting co-medications in dogs. Although two of the dogs on maintenance ASDs were also taking zonisamide and levetiracetam, and these drugs are not known inducers or inhibitors of TPM metabolism.

A limitation of this study is the small number of animals as is the heterogeneity among the dogs. However, we think our animals are more representative of the true population and allow us to preliminarily explore the effects of co-administered ASDs on TPM PK. Although our population model is based on a small number of animals, it provides useful information. We based this assertion on prior study, we did with four dogs. Using pharmacokinetic data and simulations, we were able to predict phenytoin exposure from different fosphenytoin dosing strategies and determine the optimal dosing regimen to attain the same phenytoin concentrations that are considered therapeutic in human SE in dogs (11). We subsequently used that dosing regimen to attain the targeted phenytoin concentration in a randomized safety and efficacy clinical trial (31). Based on case reports of TPM oral suspensions used to treat refractory SE, our goal target concentration range was 20–30 μg/mL. Our simulations suggest that these doses should be used in designing future a clinical trial in canine SE. Further evidence of the appropriateness of the population model is that the precision of the model parameters, and other goodness of fit criteria did not show any major model misspecifications.

Although we were only able to assess iEEG in one dog, the significant changes between pre-dose EEG energy levels and those up to 30 min after IV TPM administration suggest sufficient diffusion into the brain. This observation warrants further study in additional animals. Benzodiazepines in both rodent and dog model show significant increases in energy in frequencies greater than 4 Hz and decreases in delta frequency energy, as we saw in this one dog. These observations suggest IV TPM may be beneficial for the treatment of SE.

In conclusion, IV TPM doses of 10 and 20 mg/kg infused over 5 min were shown to be safe and tolerable in dogs. Concurrent administration of PB increased the clearance of TPM ~5.6-fold. Simulations suggest that doses of 20 and 25 mg/kg of IV TPM are necessary to achieve a target concentration between 20 and 30 μg/mL in dogs not on PB and dogs on PB, respectively. A key strength of this study is the use of animals with naturally occurring epilepsy. The results of this study provide information on optimizing TPM therapy for future studies of canine SE, which will subsequently guide the design of IV TPM clinical trials of human SE. Future work includes conducting a phase II/III efficacy study in canine SE using the dose strategy determined from the PK modeling results of this study.

## Author Contributions

IV, LC, PM, IL, GW, JC, and EP designed the study and revised drafts of the manuscript. IV, LC, and PM conducted the study, collected samples, performed data analysis, interpreted data, prepared the initial drafts of the manuscript, and performed final edits. UM developed the TPM assay and analyzed samples. DC performed the statistical analysis of iEEG data and revised drafts of the manuscript. EP also supervised the care of the animals.

## Conflict of Interest Statement

Drs. JC and IL are paid consultants for CuRx Pharmaceuticals. Dr. JC also receives payments from a licensing agreement between the University of Minnesota and Ligand Pharmaceuticals. We confirm that we have read the Journal’s position on issues involved in ethical publication and affirm that this report is consistent with those guidelines. The other authors declare no conflict of interest.

## Supplementary Material

Figure S1 **(A)** Observed versus individual predicted concentrations derived from the two compartment model using a population approach. **(B)** Observed versus population predicted concentrations derived from the two compartment model using a population approach.

Figure S2 **(A)** Visual predictive check of the population two compartment model following an intravenous bolus of TPM for a dog not on inducing co-medications. Here, the observed quantiles (red lines) are superimposed with the predictive check quantiles (black lines) over the observed data (blue circles). **(B)** Visual predictive check of the population two compartment model following an intravenous bolus of TPM for a dog on inducing co-medications. Here, the observed quantiles (red lines) are superimposed with the predictive check quantiles (black lines) over the observed data (blue circles).

Figure S3 **Electroencephalograph normalized signals from beta and low gamma frequency bands averaged over 1-min intervals in one dog**.

Table S1 **Pharmacokinetic parameter estimates estimated from a population two compartment analysis following an intravenous bolus of TPM, pooling together low- and high-dose data**. tvV, typical value of volume of distribution from central compartment; tvCl, typical value of clearance from central compartment; dCl, effect of PB presence on Cl; BSV, between-subject variability; CV%, coefficient of variation; RSE%, relative standard error.

Table S2 **Difference between energy levels averaged across each frequency band**. An asterisk (*) denotes the differences were statistically significant across all channels.

Table S3 **Differences between the averaged energy levels at 15 min pre-dose (−15 to 0 min) and 15 min post-dose (0 to +15 min) and their *p*-value (probability of having a difference that large or more extreme)**.
