# Supplementary figures and images for: Intravenous Topiramate: Pharmacokinetics in Dogs with Naturally Occurring Epilepsy
Source: Front Vet Sci. 2016 Dec 5;3:107. doi: 10.3389/fvets.2016.00107 (PMC5136567; doi:10.3389/fvets.2016.00107)

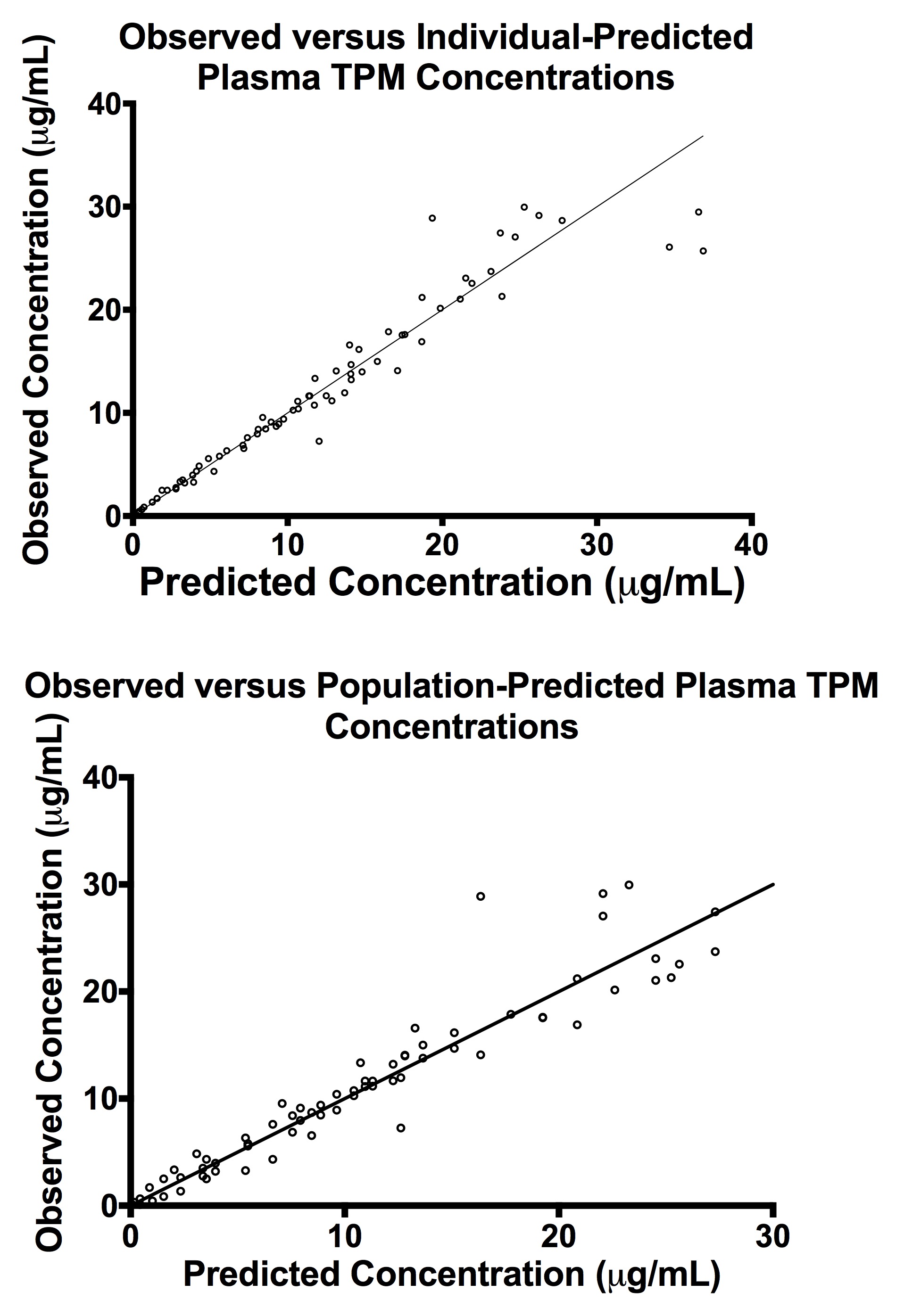

Supplement: Figure S1 — (A) Observed versus individual predicted concentrations derived from the two compartment model using a population approach. (B) Observed versus population predicted concentrations derived from the two compartment model using a population approach. [file image_1.jpeg]

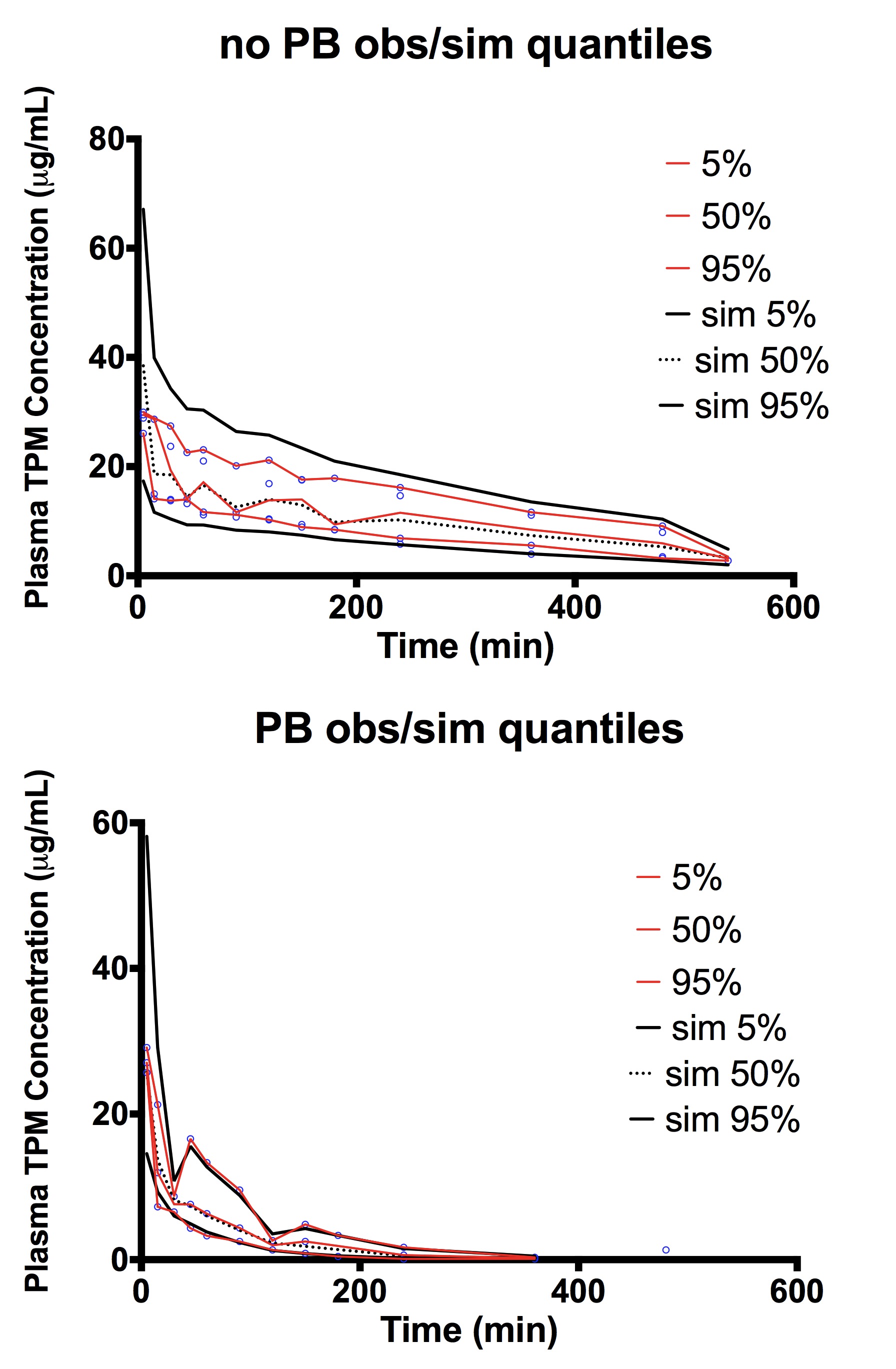

Supplement: Figure S2 — (A) Visual predictive check of the population two compartment model following an intravenous bolus of TPM for a dog not on inducing co-medications. Here, the observed quantiles (red lines) are superimposed with the predictive check quantiles (black lines) over the observed data (blue circles). (B) Visual predictive check of the population two compartment model following an intravenous bolus of TPM for a dog on inducing co-medications. Here, the observed quantiles (red lines) are superimposed with the predictive check quantiles (black lines) over the observed data (blue circles). [file image_2.jpeg]

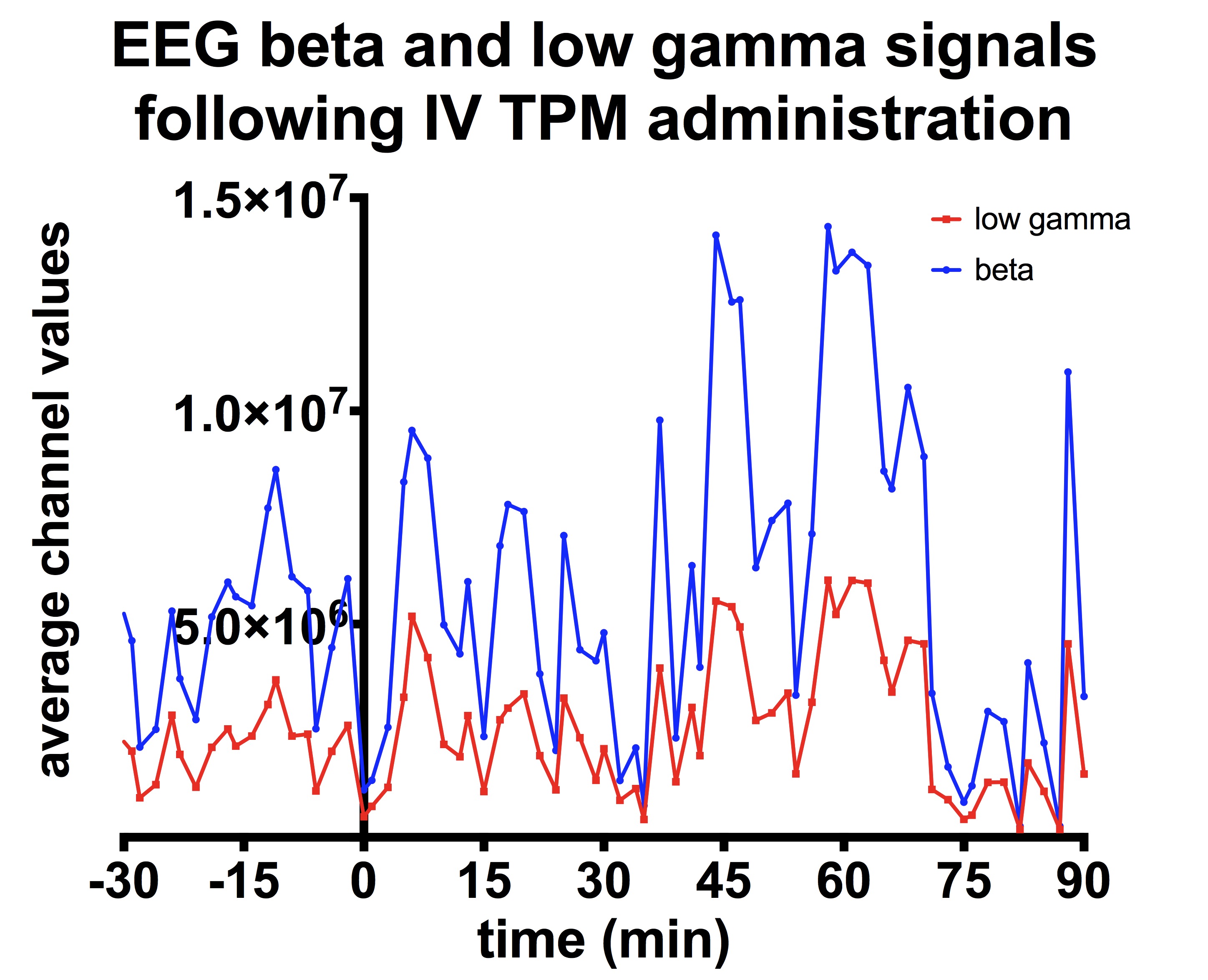

Supplement: Figure S3 — Electroencephalograph normalized signals from beta and low gamma frequency bands averaged over 1-min intervals in one dog. [file image_3.jpeg]
