# Supplementary material for: Intravenous Topiramate: Pharmacokinetics in Dogs with Naturally Occurring Epilepsy
Source: Front Vet Sci. 2016 Dec 5;3:107. doi: 10.3389/fvets.2016.00107 (PMC5136567; doi:10.3389/fvets.2016.00107)
Supplement: Table S1 — Pharmacokinetic parameter estimates estimated from a population two compartment analysis following an intravenous bolus of TPM, pooling together low- and high-dose data. tvV, typical value of volume of distribution from central compartment; tvCl, typical value of clearance from central compartment; dCl, effect of PB presence on Cl; BSV, between-subject variability; CV%, coefficient of variation; RSE%, relative standard error. [file table_1.docx]

Supplementary Material

**Intravenous Topiramate: Pharmacokinetics in Dogs with Naturally-Occurring Epilepsy**

**Irene Vuu^1,2^, Lisa D. Coles^1,2^, Patricia Maglalang^1,3^, Ilo E Leppik^2,4^, Greg Worrell^5^, Daniel Crepeau^5^, Usha Mishra^1^, James C. Cloyd^1,2^, *Edward E. Patterson^6^**

^1^Center for Orphan Drug Research, University of Minnesota, MN, United States
^2^Department of Experimental and Clinical Pharmacology, College of Pharmacy, University of Minnesota, MN, United States
^3^College of Science and Engineering, University of Minnesota, MN, United States
^4^UMP MINCEP Epilepsy Care, Minneapolis, MN, United States
^5^Mayo Clinic, Rochester, MN, United States
^6^College of Veterinary Medicine, University of Minnesota, Saint Paul, MN, United States

*** Correspondence:**Dr. Edward (Ned) Patterson
[patte037@umn.edu](mailto:patte037@umn.edu)

Supplemental Table 1. Pharmacokinetic parameter estimates estimated from a population 2 compartment analysis following an intravenous bolus of TPM, pooling together low and high dose data. tvV: Typical value of volume of distribution from central compartment; tvCl: Typical value of clearance from central compartment; dCl: Effect of PB presence on Cl; BSV: Between-subject variability; CV%: coefficient of variation; RSE%: Relative standard error

| Model Parameter | | Estimate | Units | Stderr | CV% |  |
| --- | --- | --- | --- | --- | --- | --- |
| Fixed Effect | tvV | 376 | mL/kg | 72.4 | 19.2 | --- |
|  | tvV2 | 298 | mL/kg | 56.0 | 18.7 | --- |
|  | tvCl | 1.84 | mL/(kg*min) | 0.08 | 4.52 | --- |
|  | tvCl2 | 21.0 | mL/(kg*min) | 9.37 | 44.7 | --- |
|  | dCl | 1.73 | --- | 0.13 | 7.66 | --- |
| Random Effect |  | **Estimate** |  | **Stderr** | **RSE%** | **Shrinkage%** |
|  | BSV_V_ | 0.08 | --- | 0.02 | 24.6 | 9.3 |
|  | BSV_Cl_ | 0.02 | --- | 0.01 | 53.4 | 9.18 |
|  | Residual error, CV% | 14.9 | --- | 1.71 | 11.5 | --- |
