# Supplementary material for: Intravenous Topiramate: Pharmacokinetics in Dogs with Naturally Occurring Epilepsy
Source: Front Vet Sci. 2016 Dec 5;3:107. doi: 10.3389/fvets.2016.00107 (PMC5136567; doi:10.3389/fvets.2016.00107)
Supplement: Table S2 — Difference between energy levels averaged across each frequency band. An asterisk (*) denotes the differences were statistically significant across all channels. [file table_2.docx]

Supplementary Material

**Intravenous Topiramate: Pharmacokinetics in Dogs with Naturally-Occurring Epilepsy**

**Irene Vuu^1,2^, Lisa D. Coles^1,2^, Patricia Maglalang^1,3^, Ilo E Leppik^2,4^, Greg Worrell^5^, Daniel Crepeau^5^, Usha Mishra^1^, James C. Cloyd^1,2^, *Edward E. Patterson^6^**

^1^Center for Orphan Drug Research, University of Minnesota, MN, United States
^2^Department of Experimental and Clinical Pharmacology, College of Pharmacy, University of Minnesota, MN, United States
^3^College of Science and Engineering, University of Minnesota, MN, United States
^4^UMP MINCEP Epilepsy Care, Minneapolis, MN, United States
^5^Mayo Clinic, Rochester, MN, United States
^6^College of Veterinary Medicine, University of Minnesota, Saint Paul, MN, United States

*** Correspondence:**Dr. Edward (Ned) Patterson
[patte037@umn.edu](mailto:patte037@umn.edu)

Supplemental Table 2. Difference between energy levels averaged across each frequency band. An asterisk (*) denotes the differences were statistically significant across all channels.

| Time Ranges (min) | | Frequency Bands | | | | | |
| --- | --- | --- | --- | --- | --- | --- | --- |
| Pre-dose | **Post-dose** | **delta (1-4 Hz)** | **theta (4-8 Hz)** | **alpha (8-12 Hz)** | **beta (12-25 Hz)** | **low gamma (25-40 Hz)** | **high gamma (40-120 Hz)** |
| (-15, 0) | (0, 15) | 16631.60* | 3997.93* | 1250.46* | 1214.93* | 342.61* | 265.21* |
| (-15, 0) | (15, 30) | 17765.57* | 3710.96* | 1127.09* | 972.23* | 333.26* | 220.43* |
