# Supplementary material for: Intravenous Topiramate: Pharmacokinetics in Dogs with Naturally Occurring Epilepsy
Source: Front Vet Sci. 2016 Dec 5;3:107. doi: 10.3389/fvets.2016.00107 (PMC5136567; doi:10.3389/fvets.2016.00107)
Supplement: Table S3 — Differences between the averaged energy levels at 15 min pre-dose (−15 to 0 min) and 15 min post-dose (0 to +15 min) and their p-value (probability of having a difference that large or more extreme). [file table_3.docx]

Supplementary Material

**Intravenous Topiramate: Pharmacokinetics in Dogs with Naturally-Occurring Epilepsy**

**Irene Vuu^1,2^, Lisa D. Coles^1,2^, Patricia Maglalang^1,3^, Ilo E Leppik^2,4^, Greg Worrell^5^, Daniel Crepeau^5^, Usha Mishra^1^, James C. Cloyd^1,2^, *Edward E. Patterson^6^**

^1^Center for Orphan Drug Research, University of Minnesota, MN, United States
^2^Department of Experimental and Clinical Pharmacology, College of Pharmacy, University of Minnesota, MN, United States
^3^College of Science and Engineering, University of Minnesota, MN, United States
^4^UMP MINCEP Epilepsy Care, Minneapolis, MN, United States
^5^Mayo Clinic, Rochester, MN, United States
^6^College of Veterinary Medicine, University of Minnesota, Saint Paul, MN, United States

*** Correspondence:**Dr. Edward (Ned) Patterson
[patte037@umn.edu](mailto:patte037@umn.edu)

Supplemental Table 3. Differences between the averaged energy levels at 15-minutes pre-dose (-15 to 0 minutes) and 15-minutes post-dose (0 to +15 minutes) and their *p*-value (probability of having a difference that large or more extreme).

|  | Frequency Bands (p-value) | | | | | |
| --- | --- | --- | --- | --- | --- | --- |
| Channels | **delta (1-4 Hz)** | **theta (4-8 Hz)** | **alpha (8-12 Hz)** | **beta (12-25 Hz)** | **low gamma (25-40 Hz)** | **high gamma (40-120 Hz)** |
| 1 | 1333.27 | 311.27 | 101.28 | 69.79 | 23.03 | 24.23 |
|  | 2.20E-56 | 2.92E-25 | 2.80E-33 | 2.17E-09 | 5.59E-20 | 6.20E-77 |
| 2 | 1465.80 | 351.22 | 108.98 | 92.18 | 20.74 | 20.40 |
|  | 2.13E-53 | 1.33E-32 | 7.28E-43 | 3.01E-18 | 1.65E-07 | 2.37E-26 |
| 3 | 1521.13 | 377.73 | 120.24 | 102.94 | 29.60 | 37.27 |
|  | 3.26E-54 | 1.76E-47 | 1.23E-46 | 2.77E-45 | 9.02E-47 | 2.64E-92 |
| 4 | 1433.99 | 304.59 | 103.41 | 110.60 | 33.48 | 34.39 |
|  | 3.45E-43 | 1.35E-19 | 1.00E-24 | 2.48E-50 | 6.23E-61 | 4.26E-107 |
| 5 | 1473.43 | 342.55 | 108.14 | 96.62 | 25.74 | 27.62 |
|  | 3.28E-55 | 1.61E-34 | 1.57E-39 | 6.25E-30 | 1.29E-21 | 1.48E-97 |
| 6 | 1450.46 | 343.63 | 107.91 | 100.61 | 26.36 | 23.34 |
|  | 8.61E-51 | 1.48E-35 | 1.64E-40 | 7.17E-43 | 3.19E-29 | 1.25E-80 |
| 7 | 1549.22 | 352.14 | 111.00 | 104.64 | 32.02 | 25.09 |
|  | 1.83E-52 | 3.29E-35 | 3.31E-37 | 3.16E-55 | 5.34E-51 | 1.17E-51 |
| 8 | 1438.85 | 322.78 | 106.94 | 113.37 | 35.52 | 33.77 |
|  | 6.95E-54 | 2.21E-33 | 2.64E-39 | 2.06E-65 | 3.66E-69 | 6.80E-99 |
| 9 | 244040.12 | 58780.40 | 18389.51 | 17975.24 | 5052.15 | 3804.78 |
|  | 1.00E-96 | 4.07E-86 | 8.90E-83 | 1.15E-73 | 8.41E-71 | 5.09E-57 |
| 10 | 1504.09 | 362.82 | 112.70 | 92.89 | 24.16 | 25.59 |
|  | 3.43E-48 | 3.35E-33 | 8.12E-45 | 7.71E-23 | 3.83E-19 | 1.04E-39 |
| 11 | 1480.83 | 383.10 | 114.90 | 103.88 | 31.11 | 35.00 |
|  | 1.27E-62 | 6.20E-48 | 7.80E-53 | 2.51E-56 | 6.11E-60 | 2.55E-103 |
| 12 | 1371.62 | 304.63 | 91.43 | 105.64 | 34.45 | 32.06 |
|  | 4.17E-42 | 2.69E-20 | 3.18E-25 | 4.12E-50 | 1.08E-71 | 2.72E-101 |
| 13 | 1527.61 | 354.99 | 101.35 | 61.96 | 25.42 | 32.55 |
|  | 1.52E-58 | 2.50E-28 | 1.21E-30 | 2.42E-08 | 2.26E-15 | 2.14E-82 |
| 14 | 1410.18 | 358.20 | 106.51 | 87.95 | 19.82 | 25.03 |
|  | 1.13E-58 | 3.46E-39 | 1.79E-45 | 2.95E-20 | 1.81E-08 | 1.21E-51 |
| 15 | 1579.27 | 365.92 | 113.96 | 106.58 | 32.17 | 32.11 |
|  | 3.20E-44 | 5.57E-30 | 2.77E-37 | 1.87E-57 | 8.92E-41 | 9.92E-62 |
| 16 | 1525.71 | 350.97 | 109.14 | 113.96 | 36.06 | 30.16 |
|  | 1.65E-46 | 7.40E-31 | 3.66E-39 | 4.71E-61 | 5.08E-85 | 2.54E-110 |
